# Activation of the ErbB3-AKT axis promotes melanoma cell survival and proliferation in response to RAF/MEK inhibition

**DOI:** 10.1186/1479-5876-12-S1-O2

**Published:** 2014-05-06

**Authors:** Luigi Fattore, Emanuele Marra, Maria Elena Pisanu, Alessia Noto, Claudia De Vitis, Francesca Belleudi, Luigi Aurisicchio, Rita Mancini, Maria Rosaria Torrisi, Paolo Antonio Ascierto, Gennaro Ciliberto

**Affiliations:** 1 Dipartimento di Medicina Clinica e Molecolare, Sapienza Università di Roma, Rome, Italy; 2 Dipartimento di Chirurgia “P. Valdoni”, Sapienza Università di Roma, Rome, Italy; 3 Takis srl, Rome, Italy; 4 Dipartimento di Medicina Sperimentale e Clinica, Università degli Studi di Catanzaro “Magna Graecia”, Catanzaro, Italy; 5 Istituto Pasteur Fondazione Cenci Bolognetti, Dipartimento di Medicina Clinica e Molecolare, Sapienza Università di Roma, Rome, Italy; 6 Azienda Ospedaliera S. Andrea, Rome, Italy; 7 Istituto Nazionale per lo Studio e la Cura dei Tumori “Fondazione G. Pascale”, aples, Italy

## Background

Therapy of advanced melanoma has been improved with the advent of BRAF inhibitors. However, a limitation to such treatment is the occurrence of resistance. Several mechanisms have been implied in the development of resistance, which in most cases lead to downstream MEK reactivation. In order to overcome resistance MEK inhibitors are being clinically developed with promising results. However, also in this case resistance inevitably occurs. It is commonly believed that the establishment of resistance is facilitated by adaptive changes that take place in cancer cells shortly after exposure to kinase inhibitors. Our laboratory is interested in the identification of these early adaptive changes with the intent to discover additional targets for therapeutic intervention.

## Methods

Four melanoma cell lines were tested: LOX IMVI and M14 (BRAF V600E), MST-L (BRAF V600R) and WM266 (BRAF V600D). RTK arrays (R&D) were carried out with protein extracts from untreated of BRAFi and MEKi treated cells. Western blot analysis was performed on total protein extracts using anti-ErbB3, anti-AKT and anti-ERK 1/2 antibodies. The growth inhibitory effects of multiple combinations of BRAF and MEK inhibitors and/or anti-ErbB3 mAbs were evaluated by colony formation assays. Mouse xenograft studies were carried out with M14 cells injected s.c. at the dose of 5x 10^6^ cells. Drug treatments began when tumors reached a mean volume of 100mm^3^ and tumor growth was measured by caliper.

## Results

We show that ErbB3 is the main RTK rapidly hyperphosphorylated in response to BRAF or MEK inhibition in melanoma cell lines harboring a variety of V600 BRAF mutations. This results in a strong activation of phospho-AKT. ErbB3 activation, which is caused by increased autocrine production of neuregulin, can be fully abrogated by two distinct anti-ErbB3 monoclonal antibodies, A3 and A4. Most importantly these two mAbs individually or in combination strongly enhance the ability of different BRAF/MEK inhibitors to silence the oncogenic MAPK and AKT pathways. This results in potentiation of growth inhibition and of apoptosis. Preliminary xenograft studies confirm that administration of BRAF inhibitors together with anti-ErbB3 mAbs exerts a more profound inhibition of tumor growth than single treatments which is accompanied by a stronger downregulation of oncogenic signaling.

## Conclusions

Feedback activation of ErbB3/AKT phosphorylation is a fast and common response of melanoma cells to BRAF and/or MEK inhibitors. Our results suggest that combinatorial treatment of melanoma patients with BRAF/MEK inhibitors together anti-ErbB3 antibodies should be further explored as a potentially helpful clinical approach.

